# Methacrylate Polymers With “Flipped External” Ester Groups: A Review

**DOI:** 10.3389/fdmed.2022.923780

**Published:** 2022-06-10

**Authors:** Dhiraj Kumar, Robert D. Bolskar, Isha Mutreja, Robert S. Jones

**Affiliations:** 1 Department of Surgical and Developmental Sciences, School of Dentistry, University of Minnesota, Minneapolis, MN, United States; 2 TDA Research Inc., Golden, CO, United States; 3 Minnesota Dental Research Center for Biomaterials and Biomechanics, Department of Restorative Sciences, School of Dentistry, University of Minnesota, Minneapolis, MN, United States

**Keywords:** dental, dental materials, physical properties, degradation, novel polymer, durability, methacrylate monomers, flipped ester group

## Abstract

Current resin composites have favorable handling and upon polymerization initial physical properties that allow for efficient material replacement of removed carious tooth structure. Dental resin composites have long term durability limitations due to the hydrolysis of ester bonds within the methacrylate based polymer matrix. This article outlines the importance of ester bonds positioned internal to the carbon-carbon double bond in current methacrylate monomers. Water and promiscuous salivary/bacterial esterase activity can initiate ester bond hydrolysis that can sever the polymer backbone throughout the material. Recent studies have custom synthesized, with the latest advances in modern organic chemical synthesis, a novel molecule named ethylene glycol bis (ethyl methacrylate) (EGEMA). EGEMA was designed to retain the reactive acrylate units. Upon intermolecular polymerization of vinyl groups, EGEMA ester groups are positioned outside the backbone of the polymer chain. This review highlights investigation into the degradation resistance of EGEMA using buffer, esterase, and microbial storage assays. Material samples of EGEMA had superior final physical and mechanical properties than traditional ethylene glycol dimethacrylate (EGDMA) in all degradation assays. Integrating bioinformatics-based biodegradation predictions to the experimental results of storage media analyzed by LC/GC-MS revealed that hydrolysis of EGEMA generated small amounts of ethanol while preserving the strength bearing polymer backbone. Prior studies support investigation into additional custom synthesized methacrylate polymers with “flipped external” ester groups. The long term goal is to improve clinical durability compared to current methacrylates while retaining inherent advantages of acrylic based chemistry, which may ease implementation of these novel methacrylates into clinical practice.

## INTRODUCTION

The majority of current resin based composite restorations use methacrylates as a polymer matrix. Despite clinical improvements compared to older generations, current methacrylate-based materials have less than ideal long term durability, especially in specific high risk populations (1–5). For healthy young children with a history of decay in the primary dentition, the 24 month cumulative survival rate of a methacrylate composite restoration is 67%, with no significant differences seen between the numbers of surfaces in the restoration (6). For medically complex (ASA II) children, only 55% of composite restorations survive after 24 months (6). In recent years, pediatric restorative recommendations based on systematic reviews have shifted toward recommending full coverage crowns, since current survival rates of methacrylate materials translate to primary teeth restorations failing before tooth exfoliation (7). Full coverage crowns cemented by glass ionomer cements provide a barrier seal that allows for higher success rate than multi-surface composite restorations (8). Full coverage crowns can seal carious bacteria over a long term period which allows high success rate even in the case of incomplete caries removal (9). Current pediatric restorative guidelines recommend full coverage crowns rather than composite restorations due to inherent weaknesses in the long term adhesive seal of methacrylate-based resin composite materials (7).

Laboratory assessment of current methacrylate materials have identified several weaknesses that affect long term stability of current composite restorations (10–12). Potential factors that may influence secondary caries at the composite resin tooth interface include an absence of effective buffering of methacrylate materials to acidification (13), low fluoride release of the material (14), stresses from masticatory forces (15), and degradation of the polymer matrix compromising the marginal seal and structural integrity of the restoration-tooth interface (16). While fluoride release and improving buffer capacity can be addressed with the changes to the filler system in methacrylates (17, 18), degradation is directly related to the position of ester bonds in the current methacrylate monomers. Common monomers such as ethylene glycol dimethacrylate (EGDMA), triethylene glycol dimethacrylate (TEGDMA) 1,1,1-trimethylolethane trimethacrylate, 2-hydroxyethyl methacrylate (HEMA), 1,1,1-trimethylolpropane triacrylate, urethane dimethacrylate (UDMA), bisphenol A glycidyl methacrylate (BisGMA) have internal ester bonds that irreversibly hydrolyze in the presence of saliva and oral bacteria. The hydrolysis of ester bonds severs the polymer matrix backbone structure in current methacrylates, and reduces the overall bulk mechanical properties of the material.

An obvious solution to addressing methacrylate ester bond degradation is using a replacement chemistry that shares many of the short term advantages while improving upon the long-term durability concern. Ether-based polymers have notable potential as being resistant to hydrolytic degradation (19). Vinyl sulfonamide and thiol based resin polymers show higher toughness and lower water sorption that can lead to degradation, compared to methacrylate-based resins (20). While these alternative chemistries may have reduced degradation effects through the removal of ester bonds, a comprehensive comparison to current methacrylate-based materials is still in development. In fact, one investigation into polymerization kinetics of ether-base materials required the use of the diluents EGDMA/TEGDMA, which are highly prone to hydrolysis as hydrophilic monomers (21). Methacrylate-based materials have numerous short term advantages. Methacrylates can be formulated to have favorable handling, esthetics, smell, few undesired side reactions, fast polymerization, and low temperature generation (22, 23).

Another potential solution is re-designing the methacrylate polymer linkage system. For current methacrylates, the ester bond hydrolysis that severs the internal polymer backbone leads to a reduction in bulk mechanical and physical properties. A new approach is to design monomers that when they polymerize lack ester, carbonate, acetal, and anhydride bonds within their polymeric backbone sequence.

In this brief review, recent research on repositioning or “flipping” the ester bond to an external position will be summarized. The aim of the review is to highlight the current evidence on the potential benefits of methacrylate monomers with flipped external ester groups. This review will also introduce the flipped methacrylate design as a polymer that has potential use in high-caries-risk populations such as pediatric patients where there is a need to not only improve upon outcomes for resin composite restorations, but also to increase durability for preventive sealants.

## NOVEL METHACRYLATE DESIGN AND INITIAL PROPERTIES

**FIGURE 1 |:**
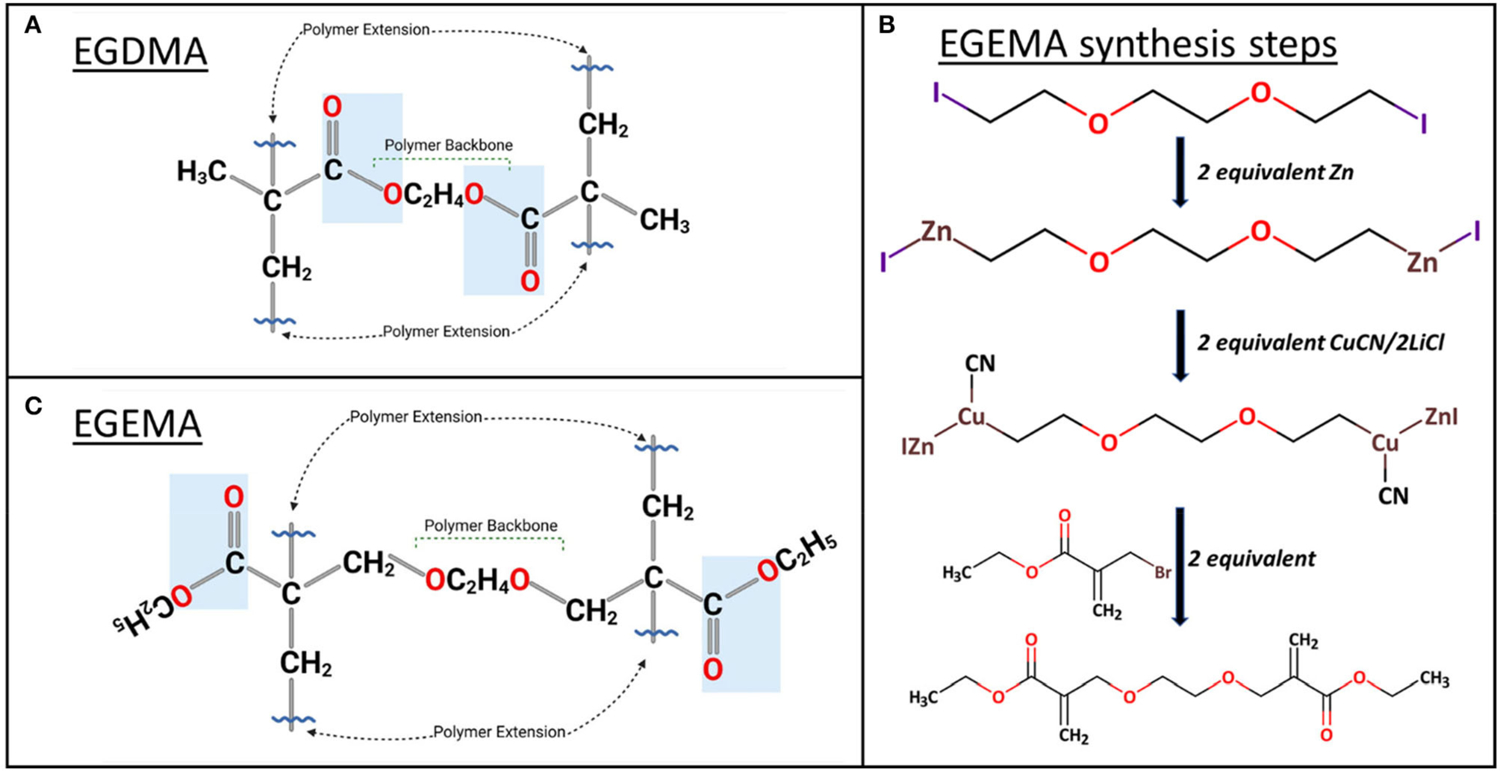
**(A)** molecular structure of EGDMA homo-polymerization where ester groups are within the polymer backbone. **(B)** EGEMA synthesis steps. **(C)** In EGEMA homo-polymerization, ester groups are external to the polymer backbone, flipped along the carbon axis, across the vinyl group.

EGDMA is used ubiquitously in dental materials. It is a hydrophilic monomer that is used as a diluent and mixed with larger strength-bearing hydrophobic monomers. EGDMA is commercially available and has been the starting point molecule for investigation into the influence of ester bond positioning. Current methacrylate monomers such as EGDMA can polymerize via a free radical mechanism mediated by a photoinitiator/co-initiators system of camphorquinone (CQ), ethyl 4-dimethylamino benzoate (EDMAB), and diphenyliodonium hexafluorophosphate (DPIHP). CQ/EDMAB/DPIHP combination has been found to be a highly efficient photoinitiator system and is activated by high-intensity light with a wavelength range of 385–515 nm (24). EGDMA can polymerize through intermolecular polymerization of vinyl groups, in the presence of other EGDMA molecules (homo-polymerization) or different methacrylate monomers (co-polymerization) (25). After polymerization, the esters within EGDMA are positioned within or “internal” to polymeric backbone sequence shown as blue boxes (Figure 1A). If hydrolysis occurs at these internal ester bond linkages, the backbone of the polymer chain is severed.

Recent studies have custom synthesized a novel molecule named ethylene glycol bis (ethyl methacrylate) (EGEMA) with the latest advances in modern organic chemical synthesis (26, 27). Unlike EGDMA, EGEMA, which is a similar molecule with an alteration in the position of the ester bonds, is not commercially available. EGEMA was synthesized in a 1-pot 2-step reaction (Figure 1B) (28). While this may indicate a straightforward synthesis, key steps in the synthesis were unavailable when the first methylacrylate dental monomers were developed (29). Briefly, the synthesis reaction, performed under argon, begins with 1,2-bis(2-iodoethoxy) ethane. This molecule is metallated with zinc and then copper using organometallic transformations (26, 27). The Zn-Cu trans-metallated intermediate is allowed to react *in situ* with two equivalents of the electrophile ethyl 2-bromomethylacrylate to generate EGEMA (Figure 1C) (28).

The main difference between EGEMA and EGDMA is the position of the ester groups in the monomer (Figures 1C,A). This translated to differences in the ester group’s relative position to the polymer backbone upon polymerization. After polymerization of vinyl groups, EGEMA has ester groups that are “external” to the polymerizing chain shown with blue box in Figure 1C. The external position can also be termed a “flipped” ester design, since EGEMA is analogous to EGDMA with the ester group flipped along the carbon axis, across the vinyl group.

Recent studies demonstrated that EGEMA and EGDMA can photo-polymerized with similar degrees of conversion after 40 second exposure to 385–515 nm high intensity (1000 mW/cm^2^) light (28, 30, 31). The two macromers used the same photoinitiator/co-initiators system of CQ/EDMAB/DPIHP. To simplify assessment of EGEMA and EGDMA, the monomers were cured into 4.9 mm diameter and 2.6 mm thickness discs within a polytetrafluoroethylene (PTFE) mold. The cured discs were initially tested for bulk mechanical properties (Table 1). These assessments indicated that EGDMA had a statistically superior hardness and diametral tensile strength (DTS) to EGEMA under the same photo-curing parameters.

### Degradation Assay Comparison

While EGDMA demonstrated initial higher mechanical properties after curing compared to EGEMA, this characterization did not predict the long term durability trend of the two materials. Degradation assays examined EGDMA and EGEMA under various aqueous storage conditions. Materials were stored in solutions with increasing hydrolytic challenge to accelerate aging and investigate the hypothesis that ester bond positioning was a primary factor in the loss of mechanical strength. In order to interpret EGEMA and EGDMA results from these various degradation assays, it is essential to review the rationale for choosing specific degradation assays used in previous studies.

While there are numerous storage solutions that tests dental material degradation potential using artificial aging models, phosphate buffered saline buffer (1xPBS) solutions at the resting saliva pH of 7.4 offers a starting point to understand hydrolysis of ester bonds. Materials can be stored at room temperature or at elevated temperatures. The advantage of elevated temperature storage is that higher temperature can accelerate material aging by increasing the rate of hydrolysis to shorten the study time (32). Material discs can be exposed to elevated temperatures which is based on an American Society for Testing and Materials (ASTM) protocol for the accelerated aging of medical devices and polymers (33). Based on ASTM F1980–16, material storage for 15 weeks at 55°C is equivalent to approximately 1 year at 37°C, which is the temperature within the oral cavity. Therefore, instead of storing material discs at 37°C for 1 year, experiments can store the materials at 55°C for 15 weeks and be expected to yield similar results. While this accelerated aging model is compatible with water or artificial saliva media, accelerated temperature aging in PBS can be compared with a second model of esterase enzymes suspended in 1x PBS (9 weeks).

An esterase storage model explores the direct mechanism of enzyme-mediated scission. There are several esterase enzymes that can be chosen for a model. There is evidence that esterases have different molecular specificity in methacrylate monomers, but the differences have been demonstrated only between molecules that have vast differences in size and hydrophobicity (12, 34). In the case of similar molecules, a Cholesterol esterase (CEase-Carboxyl ester lipase) derived from *Pseudomonas sp*. (EC 3.1.1.13) at 0.5 units/ml concentration in 1x PBS can be used. This CEase has reproducible esterase activity that is measurable with a colorimetric p-nitrophenol acetate assay (31). The CEase model is more severe for materials that contain ester bonds than the temperature accelerated model that examines effects of accelerated water hydrolysis of ester bonds. Furthermore, examining degradation effect size between a CEase and hydrolysis aging model provides direct evidence on the importance of ester bond positioning.

A third model of 9 week exposure to *Streptococcus mutans* (*S. mutans*; ATCC 700610/UA159) provides a measure of durability to an acidogenic bacterium. *S. mutans* can potentially degrade material discs via enzyme and acid formation. *S. mutans* produces a known esterase with measurably activity (30), and in previous works has shown to degrade conventional methacrylates (35–37). Acidification of *S. mutans* in Todd Hewitt media, which is supplemented with 0.3% yeast extract and 0.2% glucose, challenges material discs but also decreases the esterase activity substantially (30). In this model, fresh media is replaced daily. Esterases of *S. mutans* are more active near physiological neutral pHs (37). This is the rationale for using PBS buffer solutions within the CEase model. CEase activity is mitigated at lower pH, especially below a pH of 5 (37). Also, for any bacterial model, there could potentially be unidentified mechanisms of degradation.

**FIGURE 2 |:**
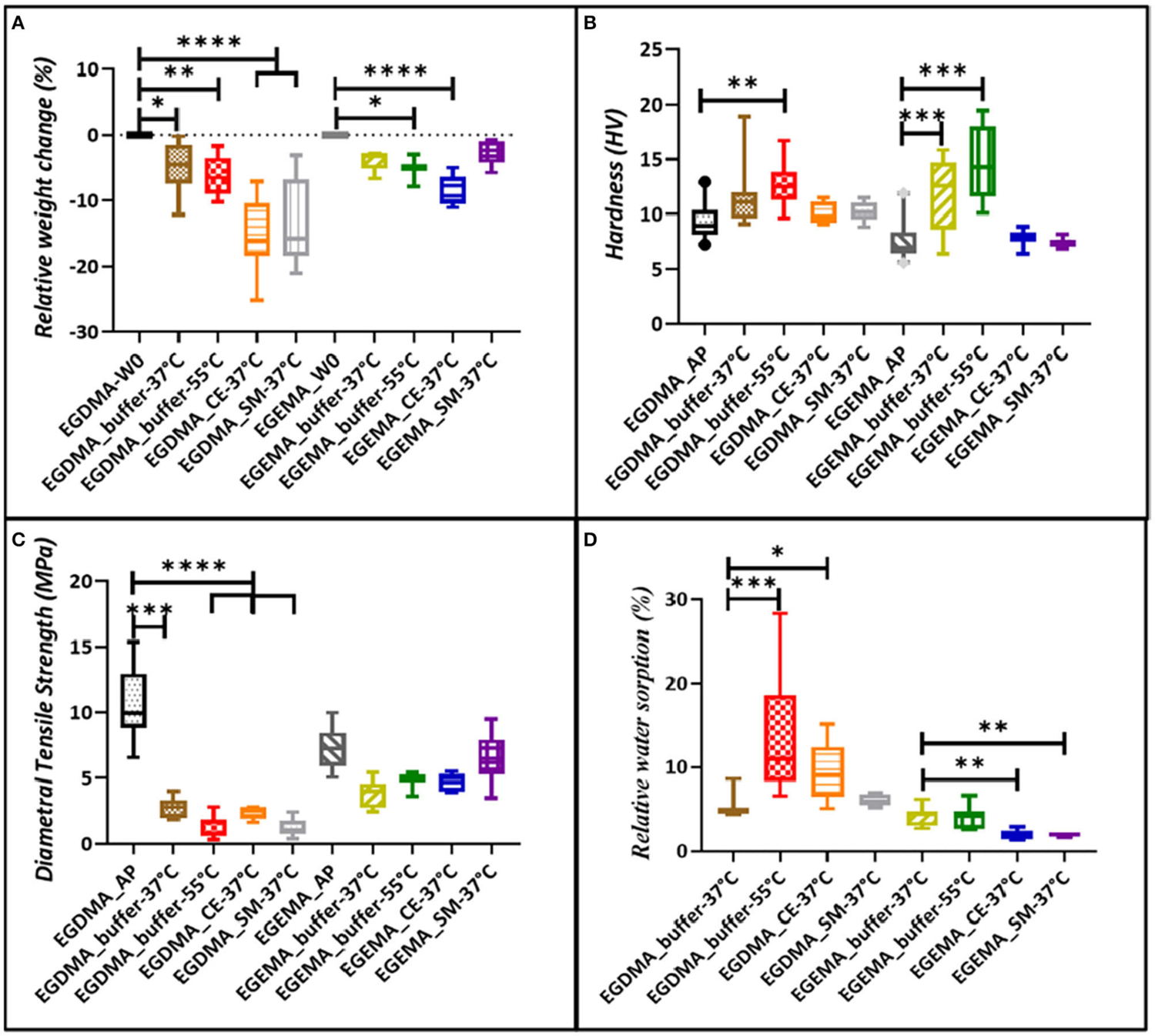
Relative weight change for sample discs incubated in buffer, CEase and S. mutans cells, **(A)** followed by change in hardness **(B)** for same conditions, **(C)** represents changes in diametral tensile strength, and **(D)** represents relative water sorption. Graphs based on data presented in reference (28, 30, 31).

In reference to studies examining EGEMA and EGDMA in these three degradation models (hydrolytic, CEase, *S. mutans*), mechanical and physical properties changes occurred for both materials (Figure 2). But the effects of degradation were more pronounced for EGDMA. EGDMA has pronounced reduction in relative weight (Figure 2A) and diametral tensile strength (DTS) (Figure 2C) when stored in the presence of buffer, CEase, and *S. mutans*. There was a trend of larger reduction in relative weight and DTS with the 55°C accelerated aging, CEase, and *S. mutans* degradation models compared to 37°C storage. In the most severe model, CEase exposure for 9 weeks, the mean relative weight loss was 15.3 and 8.8% for EGDMA and EGEMA. The relative weight loss corresponded to higher water sorption (Figure 2D). Surface hydrophilicity (lower water contact angle) increased in all three degradation assays for both EGDMA and EGEMA. Less water uptake was measured for EGEMA than EGDMA discs in all three degradation studies. The significance of these results was that material degradation increased water sorption, which in turn, mediated more ester bond hydrolysis.

EGEMA discs were more resistant to degradation effects. EGEMA had higher load-bearing DTS values after the completion of the three degradation studies than EGDMA. This shows an inversion of the differences between the two materials, since initially EGDMA had a markedly higher DTS than EGEMA.

In the three degradation studies, hardness values were less intuitive to interpret, since there were several factors happening during polymer disc storage. EGEMA and EGDMA both underwent a dark/post cure while in the storage solutions. In these degradation studies, hardness was defined as “selective intact surface hardness” since after aqueous storage hardness measurement needed to be measured on intact smooth surfaces. The testing instrumentation (Micro surface Vickers Hardness Test) was not capable of measuring hardness on irregular surface roughness caused by hydrolysis degradation. Nonetheless, the hardness results were interpreted in the context that there were two competing factors affecting surface hardness outside of the restraint of choosing intact smooth surfaces to measure. One factor was that the surface was getting harder through a dark/post cure, and the other factor was that the storage solutions (buffer, CEase, *S. mutans*) were creating surface softening though ester bond hydrolysis. It is not surprising that surface properties showed differences between the temperature accelerated aging and CEase/*S. mutans* assays.

For the temperature aging experiments, EGDMA and EGEMA disc surfaces increased, albeit slightly, in hardness. For both materials, hardness did not increase in the CEase and *S. mutans* assays. For these later studies, the surface softening was likely more aggressive and competed with the post/dark cure to a degree that no increase was seen in either material. The collective results indicate that EGEMA had surface softening from the hydrolysis of polymer side chains but degraded to a much lesser extent than EGDMA when examining the bulk properties such as weight and DTS. This is attributed to EGEMA maintaining the intermolecular bonds in the polymeric backbone sequence.

Collectively these results underscored the importance of evaluating the two materials after aging experiments rather than an initial side by side analysis that could erroneously predict better long term performance of EGDMA.

### Cytocompatibility

**FIGURE 3 |:**
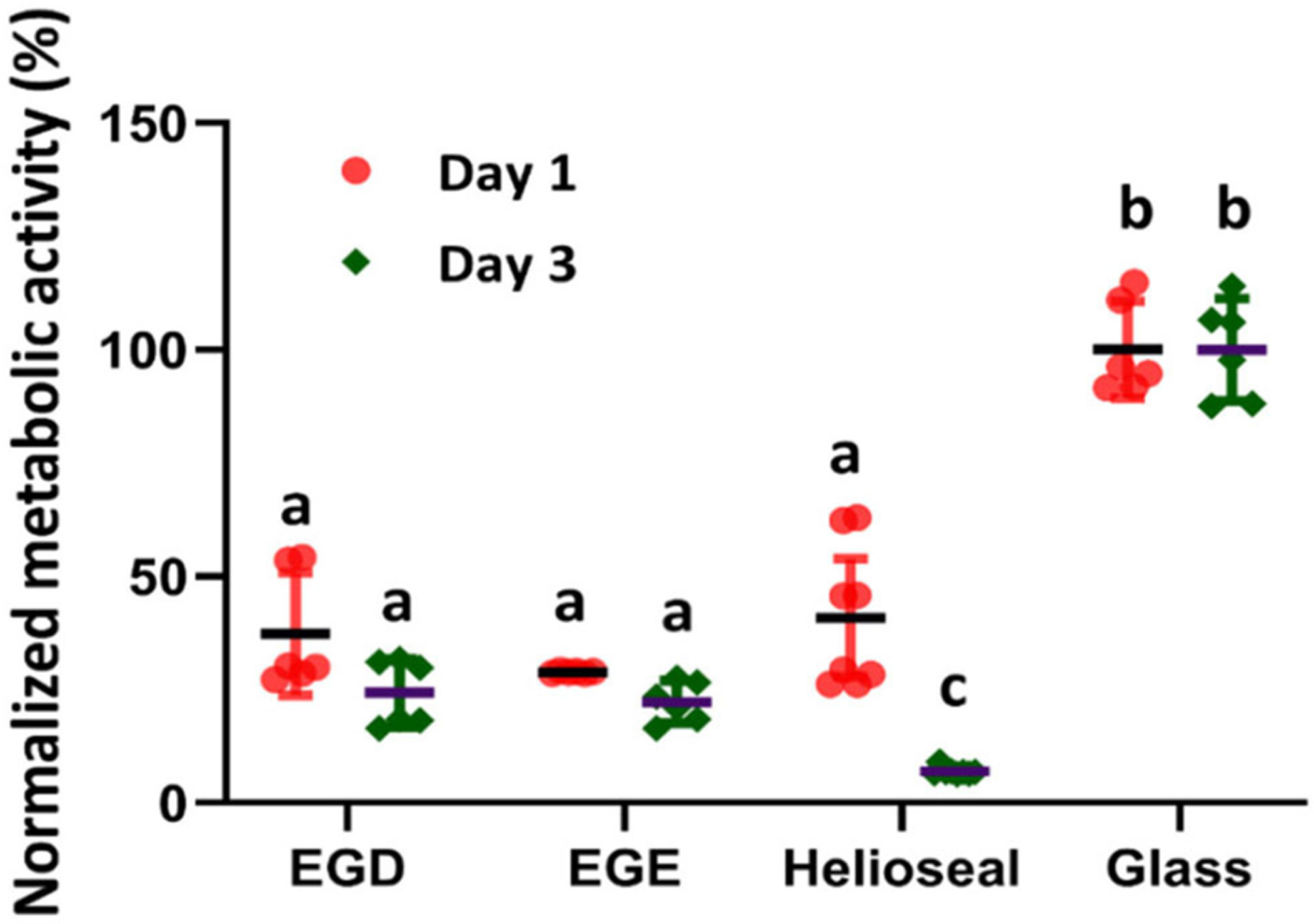
Metabolic activity of oral keratinocyte cells incubated on the EGDMA, EGEMA, HelioSeal (commercially available) discs and glass as control. Graph based on data repository reference (38).

The cytocompatibility assays of EGDMA and EGEMA indicate that both polymers have similar effects against oral keratinocytes. The data from metabolic activity assays (Figure 3), suggest both EGDMA and EGEMA support growth to a statistically significant degree more than a commercial dental sealant (HelioSeal) that is composed of a Bis-GMA and EGDMA mixture (38). This observation highlights the higher toxicity of Bis-GMA (39). EGDMA and EGEMA did not support the same level of cell growth and proliferation relative to a glass slide control. However, the glass slide control is a static surface that has limited leaching of unreacted monomers. Longer-term experiments of cytocompatibility are needed, after material aging, to determine how much the leaching of unreacted monomers contribute to the initial cytotoxicity. Prior *in vitro* studies examining traditional methacrylates have demonstrated that monomers have concentration dependent cytotoxicity and can leach from polymerized material (39–41). Whether degradation by-products also yield sustained cytotoxic effects at expected low concentrations remain unanswered but as indicated below the degradation pathways of EGEMA and EGDMA during material aging experiments are fundamentally different. As was the case with initial mechanical and physical properties, EGEMA and EGDMA may have different cytocompatibility after aging, but this has not been tested to date. Additional cytotoxicity and biological effect experiments examining EGEMA vs. EGDMA have not been performed.

### Degradation Pathways

Physical characterization and mechanical property assessment revealed clear differences in terminal weight, DTS, and relative water sorption between EGEMA and EGDMA in the various degradation assays. These measurements supported the hypothesis that polymer backbone preservation occurred in EGEMA polymerization. To test the degradation chemical reactions in EGDMA and EGEMA, the 37°C buffer and CEase assay storage solutions were analyzed for degradation by-products with liquid/gas chromatography (LC/GC)/mass spectroscopy (MS) (31).

Elucidating the unique biodegradation pathways of EGEMA and EGDMA was accomplished by integrating bioinformatics-based biodegradation predictions to the experimental results of storage media analyzed by LC/GC-MS. After samples were exposed for 15 weeks in PBS buffer solution and 9 weeks of CEase, storage solution aliquots were analyzed with chromatographic separation followed by in-line mass spectrometry. The molecular structures of EGDMA and EGEMA were also queried via the EAWAG-Biocatalysis/Biodegradation (BB) Prediction Pathway System (PPS) (42). The bioinformatics PPS created a series of degradation pathway predictions based on rules based assessment of bond scissions. The corresponding pathways create possible by-product molecules following pathways that are very likely, likely, or neutral. Based on experimental examples, degradation pathways that are considered very likely include biotransformations that occur in any biological system. In the case where the majority of bacteria contain enzymes that degrade a given bond, the definition of likely is indicated. Neutral pathways are common pathways where certain bacteria are implicated in the bond degradation. The molar mass of predicted degradation by-products could be compared to LC/GC-MS experimental results. Further details into the methodology of this approach are found elsewhere (31).

**FIGURE 4 |:**
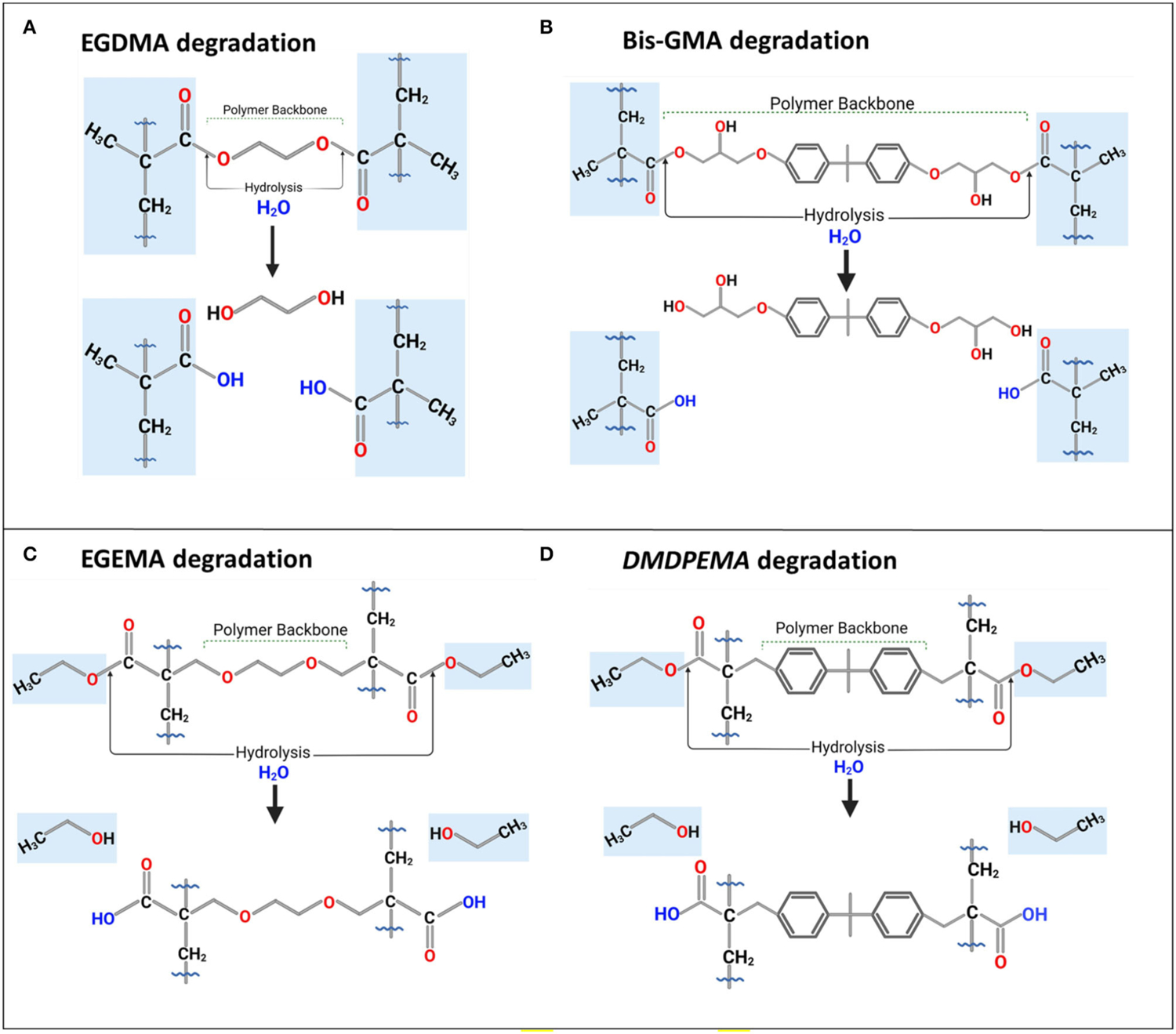
Degradation or bio-degradation of **(A)** EGDMA and **(B)** Bis-GMA with their backbone as by-products; their analogs, **(C)** EGEMA, and **(D)** DMDPEMA with ethanol as by-product and backbone preserved during hydrolysis. Figures in **(A,C)** based on figures in (28, 30, 31).

LC/MS analysis of EGDMA and EGEMA polymer discs measured 9 and 12 unique degradation by-products respectively. LC/MS analysis of EGEMA included the very likely byproducts of 2-hydroxyethyl 2-methylprop-2-enoate (HEMA) and methacrylic acid (Figure 4A). LC/MS analysis of EGEMA included the very likely by-products of 2-{[2-(3-ethoxy-2-methylidene-3-oxopropoxy) ethoxy] methyl} prop-2-enoic acid (Figure 4C).

The low molecular weight by-products of ethylene glycol, in the case of the EGDMA pathway, and ethanol, in the case of EGEMA were not identified with LC-MS. It required GC-MS analysis to identify the very likely degradation by-products of ethylene glycol and ethanol that degraded from the two polymer discs. Ethanol was found in both 37°C buffer and CEase assay storage solutions, whereas ethylene glycol was found in greater frequency in the CEase assay which might be explained by the high rate of degradation in that assay compared with 15 week buffer storage.

LC/GC-MS, integrated with a bioinformatics PPS, demonstrated that polymer backbone degradation occurred during the degradation of EGDMA, whereas EGEMA, with the external or flipped position of ester groups to the polymer backbone, had severing of side chains with the polymer backbone intact. The different ester bond positioning changed the chemical degradation pathway, by-products, and durability of the polymer discs.

## FUTURE DIRECTIONS WITH NOVEL METHACRYLATES

Altering the ester-bond linkage retains the benefits of methacrylate chemistry and the ease of commercial implementation while possibly increasing clinical longevity of resin composite restorations. Near-term investigation into substituting EGEMA for EGDMA is immediately achievable since EGEMA can be polymerized with the same photoinitiator system. Investigating EGEMA vs. EGDMA is a starting point for synthesis of many different flipped ester design methacrylate based on currently used methacrylates.

In terms of immediate improvement, the diluent EGDMA is quite hydrophilic and is likely a weak link within the polymer mixture of a resin composite system. The hydrophilicity enhances water sorption that can lead to ester bond hydrolysis. Since the synthesis reaction is worked out for EGEMA, near future work can examine EGEMA substituting for EGDMA in an EGEMA/Bis-GMA resin mixture. This EGEMA/Bis-GMA mixture can be compared with EGDMA/Bis-GMA mixture. Future work is needed to investigate subsurface degradation from water and also investigate if CEase penetration into EDGMA/EGEMA discs occurs through microcracks caused from polymerization. The degradation effects of CEase may be reduced when EGDMA and EGEMA are co-polymerized with strength bearing Bis-GMA or a flipped ester design analog of Bis-GMA.

**FIGURE 5 |:**
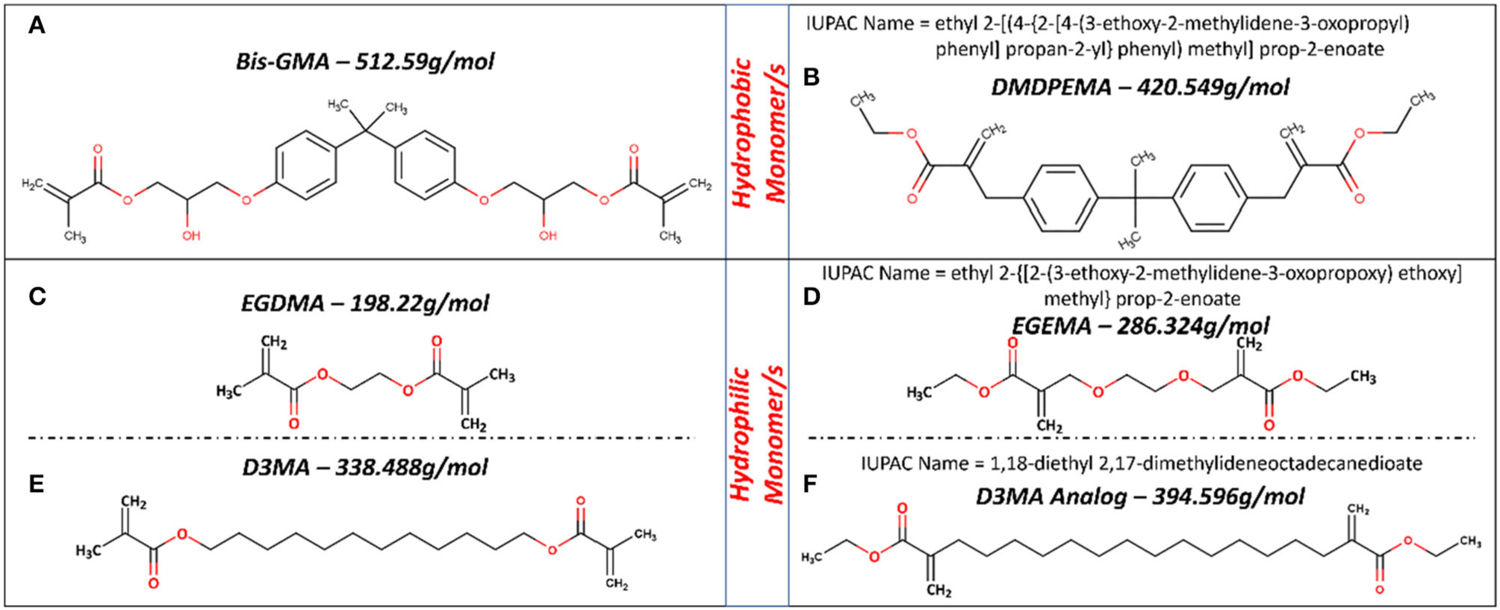
Molecular structure for hydrophobic **(A,B)** and hydrophilic **(C–F)** monomers used in dental formulations and their analog with flipped ester group design. Figures in **(C,D)** based on figures in (28, 30, 31).

Dimethyl diphenylmethane ethyl methacrylate (DMDPEMA) is flipped ester design analog of Bis-GMA that can be potentially blended with EGEMA. To achieve desirable handling properties, the addition of the low viscosity diluent EGEMA will be necessary since DMDPEMA has higher viscosity. The viscosity of DMDPEMA was not quantitatively measured yet, but it is qualitatively more viscous than EGEMA, and it is likely in the same order of magnitude as Bis-GMA. Bis-GMA needs to be blended with low viscosity EGDMA, and future work can compare DMDPEMA/EGEMA with Bis-GMA/EGDMA blends. Future work is needed to investigate DMDPEMA degradation compared to Bis-GMA degradation. Bis-GMA degradation involves breakage of the polymeric backbone yielding a main by-product of bis-hydroxy-propoxy-phenyl propane (Bis-HPPP) and methacrylic acid (Figure 4B) (43). While DMDPEMA is significantly larger than EGEMA, the external ester group can undergo hydrolysis and yield an ethanol by-product just like EGEMA (Figure 4D). DMDPEMA hydrolysis likely preserves the polymeric backbone sequence. This requires further validation with LC/GC-MS. Based on the EGEMA degradation pathway and EAWAG-Biocatalysis/Biodegradation Prediction Pathway System, all flipped ester group designs are expected to generate the same side chain hydrolysis. The main by-product is expected to be ethanol for all the flipped ester group designs no matter how large the molecule (Figure 5). The polymeric backbone is expected to be preserved in DMDPEMA and all other flipped external ester group methacrylate designed polymers. While long term immersion in high concentrations (60–75%) of ethanol can cause hydrolysis in conventional methacrylates (32, 44), the low concentration of ethanol generated from EGEMA breakdown suggests minimal chemical degradation effects (31).

The future direction of flipped ester group design polymers first requires investigation into custom synthesis of these molecules. This will be a lengthy and ongoing area of investigation. After synthesizing and purifying these flipped ester groups, investigation needs to examine how the flipped design may in some cases create steric hindrances. There may be some polymer designs that are more favorable. Future examinations will address adding branch designs that may improve shrinkage, and based on traditional ester linkage methacrylates, branching can be designed to have comparable polymerization as linear monomers (52). Importantly, shrinkage examination will require both initial testing and post-degradation assay assessment since shrinkage stress can change after aqueous conditioning (10, 45).

Current methacrylate based dental sealants have demonstrated low durability and retention (46–48). Investigating a replacement could be done with as little as two monomers, EGEMA and DMDPEMA. Once the DMDPEMA and EGEMA systems biocompatibility have been confirmed through additional *in vitro* cell studies, immunological based *in vivo* animal studies should be performed prior to human clinical trials. More work is needed to understand the biological properties and effects of methacrylate polymers with “flipped external” ester groups prior to clinical testing. For initial clinical testing, there may be benefits of optimizing dental sealants in pediatric populations as an initial clinical step. Dental sealants can be simply applied to acid etched dental enamel. An EGEMA/DMDPEMA blend is expected to be transparent to near infrared (1310-nm) light and optical coherence tomography may be utilized to assess initial sealant adaptation and longitudinal assessment of marginal integrity (28, 49).

For dental restorative materials, there are several needed steps that include optimizing the polymer matrix with filler particles and developing novel dentin bonding agents. First, while metalloproteinases (MMPs) can degrade the dentin-collagen hybrid layer, dentin bonding agents contain the same internal positioned ester bonds that can degrade EGDMA and other composites. The degradation effects of dentin adhesive hydrolysis is substantial. Even with MMP inhibitor pretreatment, water storage reduces microtensile bond strength of current adhesives and increases the marginal leakage 41% vs. initial properties (50). Cohesive failure in adhesives are also due to water adsorption and hydrolytic degradation (51). Custom synthesis of external flipped ester designs of dentin bonding agents will allow further hypothesis testing on the stability of these polymers to esterases and hydrolytic challenge. Second, these flipped dentin bonding agents may benefit from branched or dendritic designs and may have reduced water adsorption and lower esterase vulnerability than linear flipped polymers due to cross-linked shielding (52). Third, dentin bonding agents with flipped designs are likely compatible with the CQ/EDMAB/DPIHP initiator system, which was found to work in EGEMA polymerization. CQ/EDMAB/DPIHP has been previously determined to be one of the most efficient initiator systems in a moist dentin environment (24). More work is needed to directly test moisture effects with flipped ester bond polymerization. Future experiments can also use MMP inhibitors and many of the aforementioned assays and characterization to investigate how dentin bonding could be optimized. Fourth, the addition of specific filler particles to EGEMA/DMDPEMA may have beneficial effects on improving water adsorption and degradation resistance (53). Fifth, extensive mechanical and physical property testing is needed in future studies examining filled composite systems and their compatible adhesive system. Future tests include measuring the extent of chain length branching, cross-linking density, shrinkage strain, and predicted shrinkage stress (38, 54).

The trajectory of research into flipped external ester group methacrylates can test these materials in high failure risk pediatric and adult dental patients. The traditional methacrylate design was optimized in a dental healthcare system that is currently changing. Clinical outcomes, rather than application time alone, are becoming an increasingly important metric for the dental profession and insurers (55). The cost of replacing failed resin composite systems is substantial and contributes to the increase in overall annual dental expenditures. One of the main benefits of changing the approach to ester-bond linkage design is the ease of commercial implementation while improving the clinical outcome and longevity of composite resin restorations.

## Figures and Tables

**TABLE 1 |:** Initial comparison between EGDMA (internal ester position) and EGEMA (flipped) examining degree of polymerization (DoP), hardness, diametral tensile strength (DTS), and water contact angle (hydrophilicity/hydrophobicity).

	DoP (%)	Hardness (HV)	DTS (MPa)	Water contact angle
EGDMA	46.1 ± 5.53	10.9 ± 1.84	8.7 ± 2.53	58.5° ± 8.15
EGEMA (flipped)	44.9 ± 2.32	7.8 ± 1.47	6.9 ± 1.62	63.2° ± 5.34

Table based on data presented in reference (28, 30, 31).
